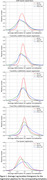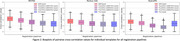# Multichannel template‐based spatial normalization precision in older adult brain QSM

**DOI:** 10.1002/alz.084791

**Published:** 2025-01-09

**Authors:** Rasheed Abid, Mohammad Rakeen Niaz, Abdur Raquib Ridwan, Yingjuan Wu, Arnold M Evia, David A. Bennett, Konstantinos Arfanakis

**Affiliations:** ^1^ Illinois Institute of Technology, Chicago, IL USA; ^2^ Rush Alzheimer's Disease Center, Chicago, IL USA; ^3^ Rush Alzheimer's Disease Center, Rush University Medical Center, Chicago, IL USA

## Abstract

**Background:**

Quantitative Susceptibility Mapping (QSM) offers significant potential for studying metal and iron homeostasis in the brain and serves as a diagnostic tool for various pathologies, such as Alzheimer’s disease. However, the precision of QSM spatial normalization for older adults depends on the quality and representativeness of the chosen template and the type of information used during image registration. This study compares three available QSM templates in terms of their representativeness and precision of inter‐subject matching for older adult QSM data.

**Method:**

The study used 3D T1‐weighted MPRAGE and multi‐echo 3D GRE data from 100 non‐demented older adults (aged 67.8‐97.2 years). QSM templates from the recently developed MIITRA templates were compared with two other publicly available templates (HybraPD and MuSus‐100). Five registration pipelines using ANTS image registration toolbox were employed. Single‐channel registration of the susceptibility maps to QSM templates, multichannel registration of T1‐weighted images and susceptibility maps to T1‐weighted and QSM templates (at 25%, 50% and 75% weights correspondingly) and finally, single‐channel registration of T1‐weighted images to T1‐weighted templates. Average log‐Jacobian maps of deformations were generated for each participant. Finally, inter‐subject pairwise (100x99/2 = 4950 pairs) normalized cross‐correlation of magnetic susceptibility maps of participants in template space was calculated for each registration approach.

**Result:**

Spatial normalization of older adult brains required less deformation when registering to MIITRA templates compared to other templates, indicating that MIITRA templates are more representative of the older adult brain (Figure 1). Furthermore, pairwise normalized cross‐correlation was higher when using single‐channel registration of susceptibility maps to QSM templates than other methods (Figure 2). Overall, the highest spatial matching of magnetic susceptibility maps of the older adult brains was achieved when using single‐channel registration of magnetic susceptibility maps to MIITRA‐QSM template.

**Conclusion:**

The study highlights the importance of template and registration pipeline selection in spatial alignment of magnetic susceptibility maps of the older adult brain. The MIITRA atlas proved more representative of the older adult brain, as evidenced by lower deformation requirements. Single‐channel registration of magnetic susceptibility maps of older adult brains to the MIITRA‐QSM template allowed higher inter‐subject spatial matching compared to other available templates.